# News and Notes

**Published:** 1902-06

**Authors:** 


### *NEWS AND NOTES.*

(We are indebted to the *Medical Age* for many of these notes.)

---

THE *Atlanta Medical and Surgical Journal* and *Southern Medical Record* having been consolidated into THE ATLANTA JOURNAL-RECORD OF MEDICINE, exchanges will please be sent only to the latter. We are getting many duplicates.

---

DR. H. S. HANSELL has received the appointment of Assistant Surgeon in the United States army.

---

THE total number of cholera cases reported in Manila up to April 13 was 245 cases with 192 deaths.

---

THE International Congress of Electrotherapy and Radiography will convene at Berne September 1 to 6, 1902.

---

DR. G. P. HUGULEY, formerly of Barnesville, has removed to Atlanta and will make this his home in the future.

---

SCARLET fever is reported in Chicago as more prevalent and more fatal than at any time during the past twenty years.

---

THE seventeenth annual meeting of the Association of American Physicians was held in Washington, D. C., April 29 and 30.

---

THE Rush Medical College will introduce coeducation in all branches of the four-years' course with the opening of the summer term.

---

THERE has been introduced in the New York legislature a bill making it a misdemeanor to employ for any other purpose bottles used for dispensing milk.

ACCORDING to an exchange, the first visit of pestilential fever to America was in 1702. It was brought from the West Indies to New York by trading vessels.

---

DR. BENJAMIN W. BIZZELL has received the appointment as Surgeon to the 5th Georgia Infantry; Dr. S. G. C. Pinckney has been appointed Assistant Surgeon.

---

A PETITION has been presented to the German Reichstag signed by the physicians of the German Empire, asking for the cremation of those dying from the plague or cholera.

---

JOSEPH J. KINYOUN, the late surgeon of the Marine Hospital Service and director of the Hygienic Laboratory at Washington, has assumed the directorship of the biologic laboratories of the H. K. Mulford Company, at Glenolden, Pa.

---

THE Fourth International Congress of Gynecology and Obstetrics will convene in Rome under the patronage of the King of Italy from September 15 to 21, 1902. Papers will be presented on the following subjects: (1) Medical indications for interrupting pregnancy; (2) hysterectomy for puerperal infection; (3) genital tuberculosis; (4) surgical treatment of cancer of the uterus. The time assigned to each paper is not to exceed fifteen minutes. Those who intend to read papers are asked to send the titles, and if possible short summaries, to the general secretary before the end of May. Membership subscription, including the right to receive printed transactions of the congress, is twenty-five lire (about $5).

---

THAT insanity is almost unknown among negroes is one of the developments arising out of the investigation into the reason for the great number of insane persons in Massachusetts institutions, it also having developed that the average of curable cases is under twenty per cent. President G. S. Hall, of Clark University, Worcester, Mass., makes this statement: "It has been observed that insanity is almost unknown among negroes. The explanation is suggested that insanity is one of the age-marks of a race, for it is certain that what the world looks on as the newer races of men, such as negroes, because of their only recent remove, comparatively speaking, from the savage state, are not susceptible to mental disorders as the white race, which has run through so many different civilizations and crucial experiences."

[This is quite at variance with the statistics of Southern insane asylums, in which the number of negro inmates is steadily increasing.—ED.]

---

IN the recent report of the cancer laboratory, University of Buffalo, Dr. Roswell Park (*American Medicine*) says that results of the past year tend to confirm the growing belief in the infectiousness of the disease, and also that it is on the increase. The year's study has embraced the problems involved in connection with parasites as a cause of cancer, and their culture on artificial media. Efforts have been made to acclimatize yeasts, fungi protozoa, etc., to development in living animals and to the foundation of tumors, and also to cultivate parasites from cancerous material on artificial media, and to facilitate their growth and development in animals. Investigations along physiologic and chemic lines have been carried out and many experiments made, especially those of a microchemic nature, with the idea of verifying the pathologic work. The examination, comparing, and indexing the work and results of others in various parts of the world have occupied much time.

---

FURTHER details of Mr. Jonathan Hutchinson's investigations of leprosy in South Africa are reported. He finds, says *American Medicine*, the disease all over the country, but common in no locality and affecting chiefly the colored races, but existing among the

Dutch farmers, among whom it was probably a new disease 150 years ago, when first recognized near Cape Town. Since that time it has spread gradually over the whole British territory, including the Transvaal and the Orange Free State. It appeared first in Natal sixty years ago, but in Zululand it is almost unknown. The disease spreads in Hottentot and Kaffir kraals, where it is generally introduced by some laborer who has brought the disease with him from Cape Town, but does not spread in leper asylums or civilized communities where care and cleanliness prevail. He considers that it is communicated by eating food contaminated by a leper's hands, and that the primary cause of the disease is the ingestion of badly-cured salt fish. He holds that the fish-canning establishments should be looked after at once.

